# A Pathological Suggestion

**Published:** 1900-10

**Authors:** John A. McClain

**Affiliations:** Philadelphia


### A PATHOLOGICAL SUGGESTION.

BY JOHN A. McCLAIN, D.D.S., PHILADELPHIA.

A CASE came to me recently which I believe will be of some interest to the readers of the INTERNATIONAL DENTAL JOURNAL, both in itself and in the suggestions which spring from it. It was that of a young lady of about twenty-five years of age.

Last November I did some work for her, and in January she came to have a large amalgam filling repaired. It was in the lower right first molar. In shaping for the repair, which involved the filling and the tooth, the pulp-canal was entered, it being a devitalized tooth, at the gingival distal buccal side, and found that the canals and chamber had been filled with cotton which was foul. The cotton was removed and the canals and chamber were cleaned, sterilized, and filled, and the filling repaired. No soreness or pain has been experienced up to this time with this tooth. Along in March she came to the office and said that she had been having trouble with that side of the jaw, but could not lay it to any particular tooth. She said there had been five swellings, each about the size of a bean, on the jaw externally. These increased in size from the front to the back of the mouth. They had disappeared when she came. I felt sure that the same tooth was involved, and suggested opening up the second and third molars, which also contained large contour amalgam fillings, for I feared that they might contain cotton fillings similar to the one I had opened, which might be the cause of the infection, and might produce more serious results. She would not consent, and I dismissed her after doing some other work. She could not tell me whether the second and third molars were devitalized or not, and I did not test them.

Her next visit was in April, to get relief from what she supposed was another abscess forming. Her face was very much swollen, the swelling even extending down the neck and to the upper part of the thorax. The parotid and submaxillary glands were involved. She could not open her mouth. I examined the mouth with the electric lamp and mirror as well as I could, inserting it through the space left where two teeth had been extracted. The tonsils were very much swollen, and all the tissues on the right side of the mouth by the lower molars, and especially posterior to them, were very much inflamed. On questioning her, she told me that the wisdom-tooth had been sore, but that had passed away. The other two had never been sore.

I wrote a note and sent her to her physician, as I considered her case one for general treatment. He treated her for nearly a week and then sent her back to me, writing me at the same time, saying, "The more I see of Miss J.'s case, the surer I am of some trouble of a septic nature that has caused the adenitis and the spasm of the masseters. Nothing that I can find explains it, and I am thrown back on the supposition that it must be her teeth. Can you find some source of infection? I do not believe that the irritation will subside until the source of the trouble is relieved. I have asked her to see you to-morrow in hopes you may find something."

The next day she came, and after another examination, I still could not see how any of the teeth was the cause, as none was sore. The soreness in the wisdom-tooth which was present at the beginning of the trouble had not continued. After consulting with a professional friend of years of experience, in whose judgment I have great confidence, I decided to take her to one of the leading oral surgeons of the city for consultation. She consented to go. He examined the case and advised the extraction of the third molar. I requested him to take the responsibility of doing this, as I did not believe that it was the cause of the trouble. When the tooth was extracted, no sign of pericemental inflammation could be found. It was as healthy-looking as any tooth could be, save that the pulp was congested. This congestion, I am persuaded, was a result of the infection and not the cause.

The next day after the tooth was extracted there was a flow of quite an amount of pus, and then followed a gradual subsidence of the swelling and a return of the tissues to a normal condition. The spasm of the masseters continued fully a week after. Where the pus came from I could not discover, although I twice tried. It did not come from the socket of the extracted tooth.

The patient told me that, at the beginning of the trouble, she had more or less fever. She suffered pain up to the time the pus was evacuated.

The second molar has recently begun to abscess, and when opened, the pulp was alive in the anterior root. I have also opened the first molar and found the canals and chamber in an aseptic condition.

This case would perhaps not have been given publicity had not the writer happened to describe it to Dr. M. G. Tull, a neighbor and practising physician. He stated that he had had three cases of infection similar to this one recently, save that there was no discharge of pus. He has kindly consented to give me a brief history of his first case, which is here given in his own words:

"Early in March, 1900, Mr. H., aged thirty years, consulted me for an attack which presented the symptoms of an ordinary case of follicular tonsilitis, there being on the surface of the tonsil a slight cheesy deposit, without marked follicular engorgement, the only pronounced feature being the great systemic prostration, which was altogether out of proportion to the local symptoms. There was no improvement on the exhibition of sodium salicylate in full dose and the local use of sodium salicylate and potassium chlorate as a gargle.

"After two days, a marked infiltration of the mucous membrane appeared, with pronounced œdema of the uvula.

"A bacteriological examination, made at the time, gave a negative result, as to diphtheritic bacilli, but Dr. Abbot personally expressed to me his opinion that the bacilli were probably present, though crowded out by other germs present. There was at no time any membrane present.

"The constitutional and local symptoms persisting for five or six days, we administered two thousand units of Mulford's antitoxin solution, which was followed by immediate subsidence of all symptoms and a recovery in forty-eight hours. The prostration persisted for weeks, and had to be met by massage, change of climate, and tonics. The case was seen with me by Dr. Van Sant."

I give the above hoping that third molars may not be uselessly sacrificed, and that the attention of both the dental and the medical profession may be turned to local infection by germs, hitherto unsuspected, in certain parts of the human body. The case which came under my observation looked to me like quinsy, or tonsillitis; but Dr. Tull regards his cases as a local infection by diphtheritic germs, unaccompanied by the usual and more marked symptoms,—the patches on the mucous membrane of the throat.

